# New Findings, Classification and Long-Term Follow-Up Study Based on MRI Characterization of Brainstem Encephalitis Induced by Enterovirus 71

**DOI:** 10.1371/journal.pone.0162877

**Published:** 2016-10-31

**Authors:** Hongwu Zeng, Feiqiu Wen, Wenxian Huang, Yungen Gan, Weibin Zeng, Ranran Chen, Yanxia He, Yonker Wang, Zaiyi Liu, Changhong Liang, Kelvin K. L. Wong

**Affiliations:** 1 Radiology, Neurology and Pediatric Intensive Care Unit Department, Shenzhen Children’s Hospital, Guangdong, China; 2 Department of Radiology, Guangdong General Hospital, Guangdong, China; 3 School of Medicine, Southern Medical University, Guangdong, China; 4 Department of Radiology, College of Medicine, University of Kentucky, Lexington, KY, United States of America; 5 School of Medicine, Western Sydney University, New South Wales, Australia; National University of Defense Technology College of Mechatronic Engineering and Automation, CHINA

## Abstract

**Background:**

To report the diversity of MRI features of brainstem encephalitis (BE) induced by Enterovirus 71. This is supported by implementation and testing of our new classification scheme in order to improve the diagnostic level on this specific disease.

**Methods:**

Neuroimaging of 91 pediatric patients who got EV71 related BE were hospitalized between March, 2010 to October, 2012, were analyzed retrospectively. All patients underwent pre- and post-contrast MRI scan. Thereafter, 31 patients were randomly called back for follow-up MRI study during December 2013 to August 2014. The MRI signal patterns of BE primary lesion were analyzed and classified according to MR signal alteration at various disease stages. Findings in fatal and non-fatal cases were compared, and according to the MRI scan time point during the course of this disease, the patients’ conditions were classified as 1) acute stage, 2) convalescence stage, 3) post mortem stage, and 4) long term follow-up study.

**Results:**

103 patients were identified. 11 patients did not undergo MRI, as they died within 48 hours. One patient died on 14^th^ day without MR imaging. 2 patients had postmortem MRI. Medical records and imaging were reviewed in the 91 patients, aged 4 months to 12 years, and two cadavers who have had MRI scan. At acute stage: the most frequent pattern (40 patients) was foci of prolonged T1 and T2 signal, with (15) or without (25) contrast enhancement. We observed a novel pattern in 4 patients having foci of low signal intensity on T2WI, with contrast enhancement. Another pattern in 10 patients having foci of contrast enhancement without abnormalities in T1WI or T2WI weighted images. Based on 2 cases, the entire medulla and pons had prolonged T1 and T2 signal, and 2 of our postmortem cases demonstrated the same pattern. At convalescence stage, the pattern observed in 4 patients was foci of prolonged T1 and T2 signal without contrast enhancement. Follow-up MR study of 31 cases showed normal in 26 cases, and demonstrated foci of prolonged T1 and T2 signal with hyper-intensity on FLAIR in 3 cases, or of prolonged T1 and T2 signal with hypo-intensity on FLAIR in 2 cases. Most importantly, MR findings of each case were thoroughly investigated and classified according to phases and MRI signal alteration.

**Conclusions:**

This study has provided enhanced and useful information for the MRI features of BE induced by EV71, apart from common practice established by previous reports. In addition, a classification scheme that summarizes all types of features based on the MRI signal at the four different stages of the disease would be helpful to improve the diagnostic level.

## 1. Introduction

Enterovirus 71 (EV71) that induces brainstem encephalitis can cause severe cardio-respiratory symptoms and even results in death [1,2]. Neuroimaging characteristics of EV71 induced brainstem encephalitis in children have been described in previous studies based on information depicted by Table 1[3–6]. The typical MRI findings are foci with prolonged T1 and T2 signal intensity at junction between pons and medulla oblongata, which pertains to the T1 weighted images (T1WI) and T2 weighted images (T2WI) that we will analyze. Previous studies did not describe in detail the variations in MR patterns and did not include large series of patients and most not employed contrast enhancement routinely in MR imaging. In fact, most other MR signal patterns have not been reported. To the best of our knowledge, we only found one study with 7 cases of follow-up MRI examinations within 2 months [4], and deduced that the diagnosis of lesions to be permanent or transient have not yet been thoroughly studied. In previous studies, findings in fatal cases have not been compared to those in recovering patients. As such, it would be valuable to learn how this disease deteriorates under the revelation of MRI.

**Table 1:** Previous studies on MRI characteristics of EV71 induced brainstem encephalitis.

	Journal	Author	Year	PMID	Number of cases	Age	Major observation in the report	MRI Follow up study	Difference from our study
1	The New England Journal of Medicine	CHAO-CHING HUANG	1999	10498488	24	<5y	MRI showed 17 of the 24 patients had high signal intensity in the brain stem on T2WI. No abnormal enhancement on T1WI	**No MRI follow-up study**.	Described only one type MR signal pattern of major lesion at acute stage
2	American Journal of Neuroradiology	Wu-Chung Shen,	1999	10588115	20	2m-7 y	15 patients had hyperintensity in the brain stem (medulla oblongata, pons, midbrain) and spinal cords on T2WI images.	2 W to 2 M: the lesions completely disappeared in five patients. The destruction in posterior medulla and pons in two patients, Most of midbrain was damaged in one patient.	No post contrast MRI study. Described only one type MR signal Pattern of major lesion at acute stag
3	Eur J Paediatr Neurol.	Chen F	2013	23561930	21	6-37m	**Type I** patch-like hyperintense T1and T2 signals restricted to the posterior brainstem in13 cases. **Type II** speckled hyperintense T1and T2 signals on vague pattern in 8 cases	No MRI follow-up study. Clinic follow-up study showed the prognosis of type II cases was better than that of type I cases.	No post contrast MRI study, Only two types MR patterns.
4	Int J Neurosci	Li J	2012	22248036	21	5-41m	21 cases of pontine encephalitis, long T1 and long T2 signal intensity was seen in the posterior portions of Brain stem.	No MRI follow up study	Described only one type MR signal pattern of major lesion at acute stage
5	Neuroradiology.	Zeng H	2012	22200974	42	5m-11y	Plain T1WI showed iso-or hypointense signals, and T2WI showed isoi-and hyperintense signals. 12 cases had slight to moderate enhancement	No MRI follow up study	Only two types MR patterns.Focus on enhancement role.
6	Neuropediatrics.	Tsai JD	2014	24258524	46		Results from viral culture and MR imaging indicated that positive identification of EV71 infection was associated significantly with lesions on MR imaging	No MRI follow up study	Described only one type MR signal pattern of major lesion at acute stage
7	Asian Pac J Trop Med.	Chen F	2013	23317889	9	4m-27m	The midbrain, pons, and medulla demonstrated abnormal signal in 3 cases.	No MRI follow up study	Only reported 3 cases
8	Zhonghua Er Ke Za Zhi.	Peng BW	2012	22801224	16	7m-5y	Bilateral hyperintense lesions, symmetrical in the posterior portions of the medulla, pons. Enhancement occurred only in the early MRI examination.	6 cased did MRI follow study	This study focus on spinal cord.
9	Int J Clin Exp Med.	Chen F	2014	25356127	12	6m-37m	MRI showed posterior brainstem abnormalities with hyperintense areas on T2WI and hypointense areas on T1WI.	No MRI follow up study	Described only one type MR signal pattern of major lesion at acute stage

This table tabulates the keyfindings of previous studies, which were compared with our study.

During an epidemics, EV71 related brainstem encephalitis is very rare and mostly occurs in the southern parts of China and in Taiwan. The interesting part is that morbidity is extremely high for patients under the age of 3 years, and can experience severe complications such as pulmonary edema, pulmonary hemorrhage, and cardiopulmonary failure. The clinical manifestations vary in terms of presentation and severity, for instance, myoclonus, ataxia, nystagmus, oculomotor palsies, and bulbar palsy presents themselves simultaneously with different degrees and combinations. The locations of EV71-induced brainstem encephalitis lesions may be detected using plain or enhanced MRI scans. Lesions based on the different stages of disease progression appears with different intensities in T1WI, T2WI, and enhanced MR images and their unique patterns can serve as a hallmark for identification of the disease stage.

Due to the very limited literature on enhanced MRI used for the diagnosis of brainstem encephalitis induced by EV71, we decided to embark on a medical study of children diagnosed with BE. In our present study, we reviewed our 3 years of clinical and medical data in consecutive cases with EV71 at a 1000 beds regional medical center that served a population of 16 million in which several epidemics had occurred since 2008. Not only did we perform long-term follow-up studies in our patients, we also took the rare opportunity to perform post mortem imaging in some fatal cases as a confirmation of our final diagnosis. According to the MRI study time point in the course of this disease, four phases were classified as 1) acute stage, 2) convalescence stage, 3) post mortem stage, and 4) long term follow-up study. The MRI signal patterns of brainstem encephalitis primary lesion were analyzed and classified according to MR signal alteration at the four stages. If an accurate diagnostics and assessment of disease severity was realized, it would help the children, especially those in the Asia–Pacific region of the world, and contribute to the health quality of mankind.

## 2. Material and Methods

### 2.1 Ethical Standards and Patient Consent

We declare that all human and animal studies have been approved by the Shenzhen Children’s Hospital ethics committee and have therefore been performed in accordance with the ethical standards laid down in the 1964 Declaration of Helsinki and its later amendments. We declare that all patients gave informed consent prior to inclusion in this study.

### 2.2 Ethics Statement

This study was approved by the Ethics Committee of Shenzhen Children’s Hospital and written informed consent was obtained from parents/guardians for minors in this study. This was cohort study and met its criteria. STROBE Statement—Checklist of cohort study was filled and submitted.

Clinical and neuroimaging data of 91 critical pediatric hand foot mouth disease (HFMD) and/or herpangina cases complicated with brainstem encephalitis were retrospectively analyzed. All patients were confirmed with EV71 infection, and were then hospitalized in Pediatric Intensive Care Unit (PICU) in Shenzhen Children’s Hospital, China, from March 1st, 2010 to October 31st, 2012. All patients were examined by a board-certified pediatric neurologist. For this study, 31 of the 91 patients were randomly recalled to undergo follow up MRI study during the period from December 2013 to August 2014.

Stage methods: the first two weeks after onset of the disease was clinically defined as acute stage, and after that it was set as the convalescence stage. Postmortem stage, plain MRI scans were performed immediately after death in two fatal cases. Parental consents were obtained from their parents before MRI scan. Follow up stage, 31 patients did the flow-up study as previous described.

### 2.3 Clinic data

Between March 1st, 2010 to October 31st, 2012, 217 children were hospitalized in Shenzhen Children’s Hospital, Shenzhen, China, to treat HFMD and/or herpangina infected by EV71. Of these subjects, 103 patients were admitted into PICU because of neurological complication of brainstem encephalitis. Among these patients, 13 of them deteriorated rapidly and quickly died within 48 hours of hospitalization, which resulted in a lack of chance to perform MRI diagnostics. Nevertheless, 2 of them underwent brain MRI scan immediately after death when parental consent was obtained. Another 1 subject died on the 14th day of the course, we did not have chance to perform MRI exam on this child. Therefore a total of 91 patients were consecutively enrolled, including 54 males and 37 females with average age 2.6(±1.9) years, ranging from 4 months to 12 years. Table 2 illustrates the involved children demographics in this study.

**Table 2:** Results of demographics data, clinic data and MRI findings.

Stage	Classification	Number	Percentage in Stage	Age(M±Std)	Gender		Lesion Predominant MR features					clinical distinctive feature	Follow-up Number	Positive Follow up result
					M	F	shape	T1WI	T2WI	Enhance	Figure			
Acute stage	I	25	32.89%	2.61y (4m-7.1y)	14	11	Fleck	Hypo-	Hyper-	Non	Fig 2	Myoclonus, tremor, and ataxia, dysfunction of cranial nerves	11	2 cases belongs to follow-up type I, 1 belongs tofollow-up type II
	II	15	19.74%	2.63y (9m-6.2y)	8	7	Fleck	Iso/Hypo-	Hyper-	Yes	Fig 3	Myoclonus, tremor, and ataxia, dysfunction of cranial nerves	6	1 case belongs to follow-up type I
	III	10	13.16%	2.40y (11m-5Y)	6	4	Fleck	Neg-	Neg-	Yes	Fig 4	Myoclonus, tremor, and ataxia, dysfunction of cranial nerves	3	
	IV	4	5.26%	1.09 (8m-2.1y)	3	1	Fleck	Neg-	Hypo-	Yes	Fig 5-	Myoclonus, tremor, and ataxia	1	
	V	2	2.63%	2.55y (2.3y-2.8y)	2	0	Whole Brainstem	Hypo-	Hyper-	Non	Fig 6	Comma, neurogenic pulmonary edema	1	
	Negative	20	26.32%	3.15y(9m-12y)	11	9	—	Neg	Neg	Non	-	Myoclonus, tremor	6	
Convalescence stage	A	4	30.77%	1.89y (8m-3.1y)	2	2	Fleck	Hypo-	Hyper-	Non	Fig 7	Neurogenic pulmonary edema	1	1 case belongs follow-up type II
	B	9	69.23%	2.11y (6m-3.6y)	6	3	—	Neg-	Neg-	Non		Myoclonus, tremor	2	
										**FLAIR**				
Postmortem stage		2		2.60y (2.2y-3y)	2		Whole brainstem	Hypo-	Hyper-	Hyper-	Fig 8	Coma, respiratory and circulation failure		
Follow up	Ⅰ	3	9.68%	5.36y (3.9y-6.7y)	2	1	Fleck	Hypo-	Hyper-	Hyper-	Fig 9	Neurogenic sequeala		
	Ⅱ	2	6.45%	4.95y (4.4y-5.8y)	2		Fleck	Hypo-	Hyper-	Hypo-		irregular breath		
	Ⅲ	26	83.87%	5.62y(3.7y-8.3y)	15	11	—	Neg-	Neg-	Neg-				

This table summarizes the clinical data and classifies the MRI features

Throat swabs and stool specimens were collected from each patient for the detection of EV71 by using reverse transcriptase polymerase chain reaction (RT-PCR). Brainstem encephalitis was clinically diagnosed according to neurological symptoms associated with the brainstem function, including myoclonus with tremor and/or ataxia, myoclonus with cranial nerve involvement, and/or rapid cardiopulmonary failure. Although neuroimaging could support the diagnosis at that time, it was not a necessary criterion [7]. Clinical manifestations and symptoms were reviewed and analyzed. Acute stage was defined as disease infection within the first two weeks and convalescence stage was after this stage.

### 2.4 Magnetic resonance imaging study

#### 2.4.1 Initial MRI study

All patients (89 cases) underwent pre- and post-contrast cranial MRI scans using the Signa Excite 1.5T imaging system (General Electric, Fairfield, USA). Pre-contrast scan included axial T1-weighted image (T1WI), axial T2-weighted image (T2WI), axial fluid-attenuated inversion recovery (FLAIR) and diffusion weighted imaging (DWI), and sagittal T1WI. Post-contrast scan included axial and sagittal T1WI. Thickness/spacing was 6.0/2.0 mm for all axial slices, and was 3.5/0.5 mm for all sagittal slices. Sequence parameters were as followings: T1WI employed T1-FLAIR sequence (TR = 2,307ms, TE = 10.6ms, TI = 620ms), T2WI used FRFSE-XL (TR = 4,000ms and TE = 104.4ms), FLAIR sequence parameters were TR = 8,420ms, TE = 127ms, and TI = 2,100ms, EPI DWI (TR/TE 6,000/82 ms, b-value = 800). Post-contrast T1WI employed a FSE sequence (TR = 500ms andTE = 20ms). The MRI contrast agent was gadopentetate dimeglumine (Bayer, Germany). Our injection dosage was 0.1 mmol/kg. Uncooperative patients were sedated with chloral hydrate enema (0.6 ml/kg) before the MRI scan.

#### 2.4.2 Follow up MRI study

In 31 cases were performed on the 3.0T Skyra MRI (Siemens, Germany). Scan sequences included axial T1WI andT2WI, sagittal T1WI and FLAIR. T1WI employs T1_FLASH (Fast Low Angle Short) sequence, and T2WI employs T2_TSE sequence, FLAIR employs a t2_tirm_tra_dark-fluid. The parameters of above sequences were as follows: Axial and sagittal T1WI (T1_fl2d_tra,TR = 244ms, TE = 2.93ms, Flip angle = 70, Average = 2, Concatenation = 1, Distance factor = 20); Axial and sagittal T2WI (TSE, TR = 3,800ms, TE 112, Average 1, Concatenation 2, Flip angle 150, Turbo factor 19); axial FLAIR (t2_tirm_sag_dark-fluid TR = 900ms, TE = 81ms, TI = 2,500ms, Flip angle 150, Distance factor = 20, Average = 1, Concatenation = 2). For all axial images, scan thickness was 3mm, and distance factor was 20%. For all sagittal images, the implemented thickness was 3mm, and the distance factor was 15%.

#### 2.4.3 MRI findings classification method

In this study, we focused on the MRI signal alteration of the primary lesion in the brainstem encephalitis. To the best of our knowledge, this has not been thoroughly investigated, and the medical community normally took it for granted that prolonged T1 or prolonged T2 signal were constant throughout the entire disease infection, which was mainly due to the limited cases for conclusion. However, from the records of our rare patient database over the two years, primary lesion MR signal changed along with the disease development. According to the course of disease, classically three stages were defined, acute stage (from onset to 14th day of disease), convalescence stage (after the 14th day of this episode) and long-term flow up (called back follow-up study). Some patients were very critical, and did not have a chance to do the MRI exam at acute stage. Therefore, in this study, MRI classification of the primary lesion of brainstem encephalitis mainly based on the signal change and disease stages. Additionally, post-mortem studies were performed on two fatal cases. The main pathology changes were inferred from the MR signal, inflammation or hemorrhage. Two experienced pediatric radiologists, who were blind to the clinic results, were introduced to evaluate and classify the MRI findings independently. If there was a disagreement, they were asked to discuss and to make a final consensus.

## 3. Results and Findings

From a total of 103 patients, 91 patients with MRI data were included in this study.

### 3.1 Clinical manifestations and symptoms

At the acute stage, fever, maculopapular lesions and blisters on the hand, foot, mouth, and buttocks were the most common clinical manifestations. The three major neurological manifestations were myoclonus, tremor, and ataxia. 42 (46%) cases pertain to the involvement of cranial nerves (VI, VII, IX, X, XI, and XII). The dysfunction of cranial nerves were facial palsy, ophthalmoplegia, dysphagia, oculomotor palsy, et al. Mental status change varied from drowsiness, lethargy, to coma. 19 (21%) patients experienced a different extent of fulminant pulmonary edema and/or pulmonary hemorrhage. The fulminant pulmonary edema was also called neurogenic pulmonary edema as the lesion damaged the respiratory and circulation control center in medullar oblongata.

### 3.2 MRI findings and classification

**Fig 1:**
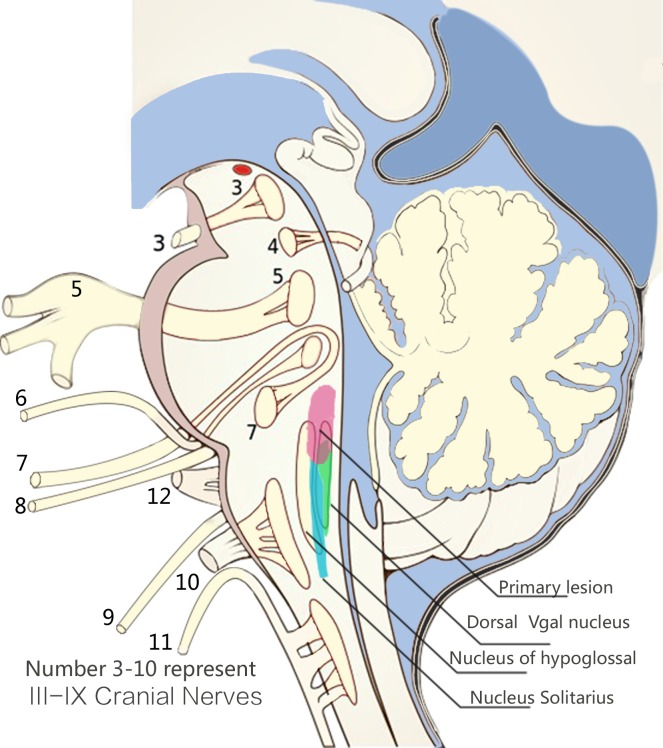
Loctation of primary lesion. The diagram showed the location of primary lesion of brainstem encephalitis induced by EV71, which was a longitudinal lesion at the posterior junction region of the pons and medulla oblongata.

The primary lesion of brainstem encephalitis that was induced by EV71 was a longitudinal fleck, which located at the posterior junction region of the pons and medulla oblongata (Fig 1). The alterations of MR signal of primary lesion varied greatly due to difference course and severity of the disease. The MRI findings of primary lesion were summarized in Table 2, and the typical MR images of each type were presented to help readers understand this concept clearly.

### 3.3 MRI signal patterns in four stages

All patterns in the four stages of the disease were scientifically catalogued, which were summarized in Table 2. In our paper, the use of enhancement pertains to the implementation of contrast agent to develop a post- enhanced image that can reveal the lesion with a higher definition and clarity. In particular, we present more explanations as follows:

#### 3.3.1 Acute stage

**Fig 2:**
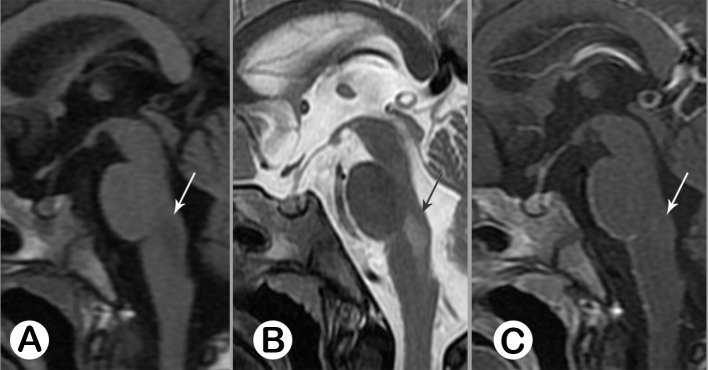
Acute stage typeⅠ.Female, 3 years old, on the 9th day of the disease. Sagittal MR images revealed a longitudinal lesion at the posterior junction region of the pons and medulla oblongata, with prolonged T1 (A, white arrow) and prolonged T2 (B, black arrow) signal, without enhancement (C, white arrow). See follow-up study in Fig 9.

**Fig 3:**
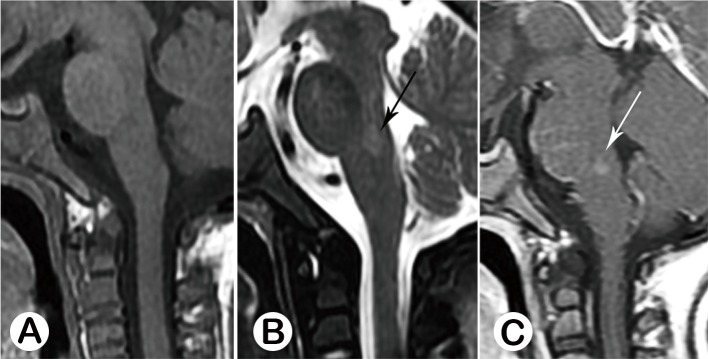
Acute stage type Ⅱ. **Male 20 months old. Sagittal T1WI (A) appeared normal**. A patchy lesion with prolonged T2 signal was revealed on sagittal T2WI (B, black arrow), with mild enhancement (C, white arrow).

**Fig 4:**
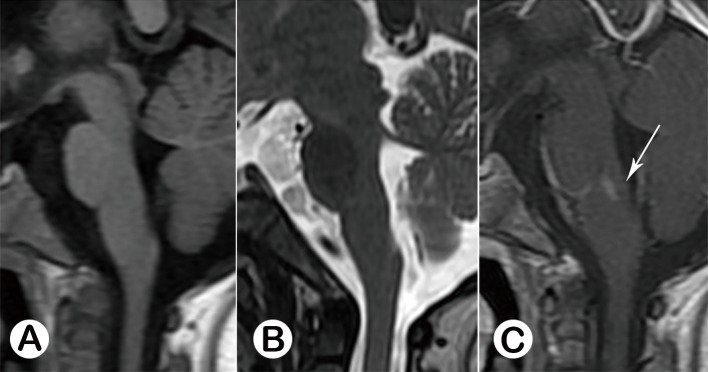
Acute stage type Ⅲ, Female, 2.5 years old, on the 7th day of the disease. There was no abnormal signal at the brainstem seen from sagittal T1WI (A) and T2WI (B); Post-contrast sagittal T1WI demonstrated a patchy, moderate enhancement region (C, white arrow).

**Fig 5:**
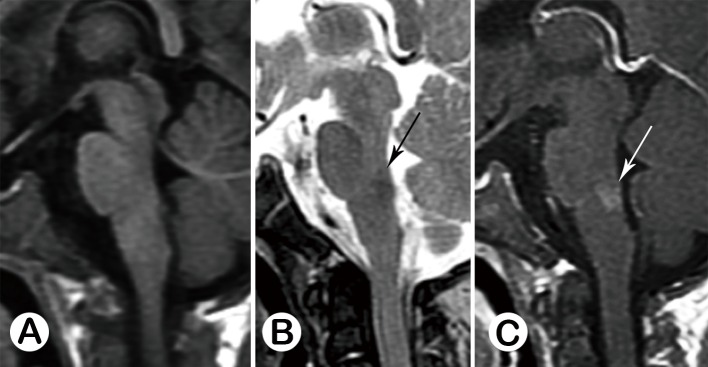
Acute stage type IV, Male, 6 months old, on the 6th day of the disease. There was no abnormal signal at the brainstem seen from sagittal T1WI (A). T2WI showed a hypo-intensive lesion at the posterior junction region of the pons and medulla (B, black arrow), which had moderate enhancement (C, white arrow).

**Fig 6:**
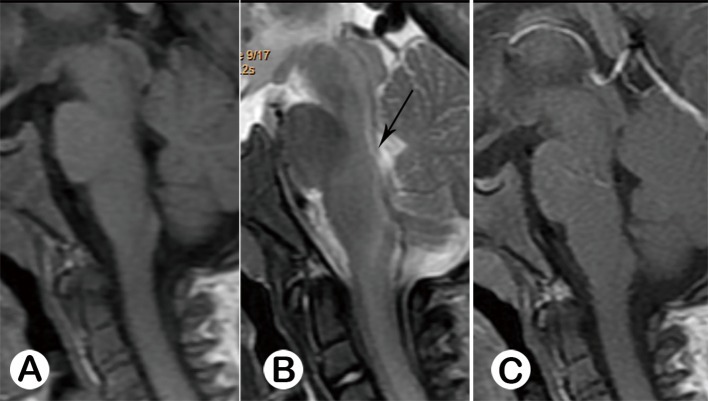
Acute stage type V, Male, 28 months old, on the 11th day of the disease. Whole brain stem was involved in this case, from midbrain to medullar oblongata, demonstrating as prolonged T1 (A) and prolong T2 signal (B, black arrow), without enhancement (C). This subject was hospitalized in PICU for 20 days, and final discharged. Follow-up study showed that there were neurologic sequealae, presenting as irregular breath and extremities tremor during sleeping.

The primary lesion signal alterations consisted of 5 patterns, which were listed in Table 2 and summarized as follows: 1) Prolonged T1 and T2 signal, without enhancement, (*n* = 25, 32.9%); 2) Prolonged T1 and T2 signal, with enhancement (*n* = 15, 19.7%); 3) Negative on T1WI and T2WI, but having enhancement (*n* = 10, 13.2%); 4) Normal on T1WI, Low intensity on T2WI, with enhancement (*n* = 4, 5.3%); and 5) Involvement of whole brainstem, with prolonged T1 and T2 signal (*n* = 2, 2.6%). (Figs 2–6). Point 4) was novel findings while compared with previous study in Table 1.

#### 3.3.2 Convalescence stage

**Fig 7:**
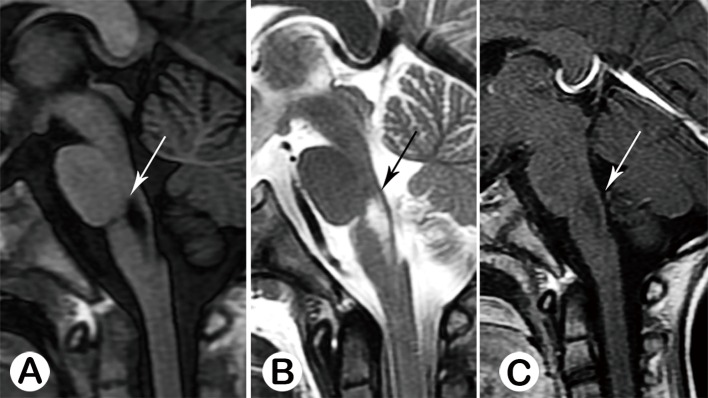
Convalescence Stage, Male, 4 years old, on the 28th day of the disease. MR images showed there is a longitudinalmalacia cavity at the posterior junction region of the pons and medulla, with prolonged T1 (A, white arrow) and prolonged T1 (B, black arrow) signal, without enhancement (C, white arrow). This subject had had impaired function of breath and recurrent pneumonia after discharge.

The primary lesion of 4 cases displayed as prolonged T1 and T2 signal, and without enhancement (*n* = 4). The other 11 cases had no abnormal MRI finding. (Fig 7)

#### 3.3.3 Post-mortem stage

**Fig 8:**
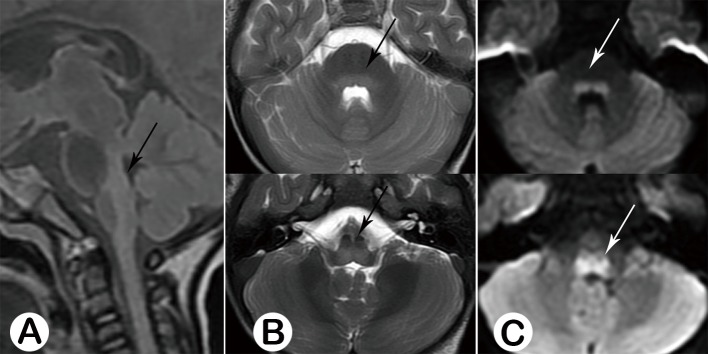
Postmortem stage, Male, 37 months old, on the 2nd day of the disease. **Sagittal** FALIR (A, black arrow) and axial T2WI (B, black arrow) showed the whole posterior portion of the brainstem was involved, demonstrating hyper-intensive signal, with restricted diffusion (C, black arrow).

MRI findings revealed the posterior portion of whole brainstem that was involved with prolonged T1 and T2 signal, with restricted diffusion in 2 cases. (Fig 8) This was also a novel finding.

#### 3.3.4 Long-term follow up MRI study

**Fig 9:**
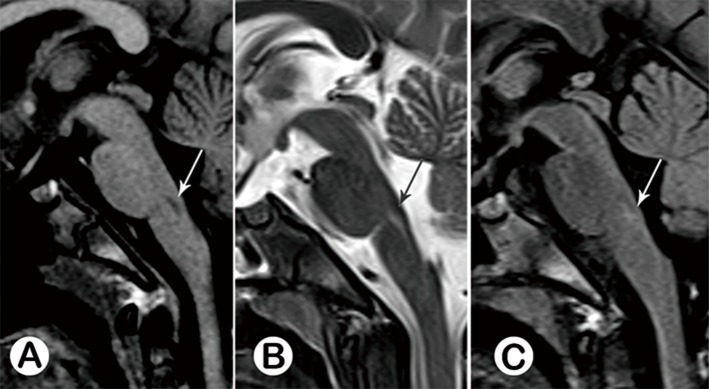
Follow-up study of previous case (Female, 6 years 8 months old,from Fig 2) in 3 years 8 months. Sagittal MR images revealed the lesion was smaller while compared with Fig 2, demonstrating prolonged T1 (A, white arrow) and prolonged T2 (B, black arrow) signal, and hyper-intensive signal on FLAIR(C, white arrow). This subject had special neurological sequelae, which were irregular breath and involuntary extremity tremor during sleeping at night.

26 cases (84%) were negative. Smaller lesions at their original place with prolonged T1 and T2 signal in 3 cases, hyper-intensity on FLAIR. 2 of the 3 cases’ MRI signal belonged to Type I, and one belonged to Type II in acute stage. MRI findings of these three cases suggested that it was gliosis change. Also, malacia cavities at previous lesion region were found in 2 patients, which belonged to Type I in acute stage and Type A at convalescence stage separately. These five patients had special neurological sequelae, including irregular breath and involuntary extreme tremor during sleeping at night. (Fig 9)

## 4. Discussion

In the study, the clinical manifestations of 91 critical cases with brainstem encephalitis induced by EV71 from PICU were summarized. Also, MR findings of these cases were thoroughly investigated; classifications were also made according to phases and MRI signal alteration. To the best of our knowledge, after comparing our findings with previous reports (see Table 1), ours is the most comprehensive study with the world’s largest number of cases. Furthermore and most importantly, we have focused on detailed information of MR signal alteration, made relevant and useful classification, as well as validated and confirmed the clinical applicability of our scheme using a long-term follow up study.

### 4.1 Clinic manifestations

EV71 has been proven to be a causative agent of severe and fatal cases in several large outbreaks of HFMD or herpangina in Asia [2,7]. A small portion of the patients especially those in the critical condition tend to have neurological complications, including aseptic meningitis, acute flaccid paralysis, and brainstem encephalitis. According a recent review report, brainstem encephalitis was the most common neurological complication, accounting for 58%, followed by aseptic meningitis [7]. Brainstem encephalitis, which can cause neurogenic pulmonary edema/hemorrhage and cardiorespiratory failure, or even fatality, was considered as the most critical neurological complication, drawing great attention from the medical community[1]. Results of multivariate analysis indicated RR.26/min, Age<4yo, GLU.8.3>mmol/L, LYM.>40% were risk factors [8]. In our present study, the average age was 2.6 (±1.9) years, 54 were males, accounting for 59%. The demographics features were consistent with the other studies in Asian countries and regions [8–10]. The most common neurological manifestations of brainstem encephalitis induced by EV 71 were myoclonus and tremor in this research, which were similar to the other studies [1,11,12]. 19 (21%) patients complicated a fulminant pulmonary edema; the incident rate was lower than previous study in our hospital located within Shenzhen, and also in Taiwan. This was mainly because of the valuable experience learned from an EV71 outbreak in 2008 by applying positive end-expiratory pressure ventilation in the early phase of disease infection.

### 4.2 Pathology and MRI findings

Prior to discussion of the MRI findings, it is important to review the histopathology change revealed by previous studies for a better understanding of basis behind the MRI. Autopsy demonstrated that inflammation in the central nervous system (CNS) was most severe in the gray matter of spinal cord and brain stem. In our present study, the case presented in Fig 6 and the postmortem case in Fig 8, where MRI showed that the brainstem was seriously affected. The affected neurons were mostly destroyed and infiltrated by inflammatory cells [13]. Autopsy studies revealed that histopathology changes included neuronal degeneration and necrosis, neuronophagia, perivascular cuffing and diffuse or nodular hyperplasia of macrophages/microglia, which were caused by virus neurotropism rather than by ischemic injury [13,14]. Previous study showed that EV71 virus can be isolated from different CNS tissues, including the pons, medulla, cerebellum, and spinal cord [13]. EV71 antigen was positive in the cerebella and brainstem, evidence from immunohistochemical aspect. Researchers also found that viral loads were highest in brainstem among all tissues [14]. There are accumulating evidence support that EV71 can invade the brainstem directly and cause brainstem encephalitis, especially those with respiratory distress, cyanosis, poor peripheral perfusion, shock, coma[1,13]. Another study pointed out that cytokines, lymphocytes and monocytes contribute significantly to pathogenesis [15].

The MRI findings and classification based on three stages were discussed. Some of MRI findings of brainstem encephalitis induced by EV71 have been reported by many studies [1,4–6,11,16]. The prominent MRI feature of brainstem encephalitis was the location of primary lesion, which was set on the posterior of the pons and medulla oblongata. The most common alteration of MRI signal was prolonged T1 and T2 signal of the lesion. The figures in Table 2 of our study also confirmed this finding. Studies also demonstrated that in some cases, the lesion can be enhanced on post-contrast MRI scan [5,6]. Our previous study recommended that routine pre- and post-contrast MRI was an optimal choice because this method can reveal the lesions that might be unnoticed in plain MRI scan [5]. In this study, a new type (Type IV) signal alteration in acute stage was reported, presenting as normal on T1WI and hypo-intensive on T2WI and having enhancement. The average age of the type was 1.09 (8m-2.1y), which was much lower than other group. Combining the pathology change from the above paragraph and MR signal, the possible explanation for hypo-intensity on T2WI might be that intracellular deoxyhemoglobin in red blood cell occurs in acute stage (1–3 days). There are about 25% patients (20 of 76) with negative MRI results in acute stage. The MRI signal patterns in convalescence stage in this investigation are similar to our previous report[5]. Here, 5 types of the MR signal patterns in acute stage could help the pediatric radiologists to achieve a clear map of the signal variation. It was also necessary to be reminded that brainstem encephalitis was diagnosed clinically and a MRI negative result was not an exclusive criterion. As the pediatrician make the diagnosis of the EV71 related brainstem encephalitis, especially for those who were hospitalized in PICU, it is extremely important and necessary for physician to have clear image of the brainstem, to what extent the brainstem was involved. Furthermore, both our previous study [5] revealed that there was positive correlation between MRI scores and clinic scores. Plusing the results from this study, it was very safe to say that MRI plays a very important role in the evaluation of the EV71 related brainstem encephalitis, not just for the diagnosis but also for the treatment and prognosis prediction.

To the best of our knowledge, this was the first report on postmortem stage MRI findings. From 2 fatal cases, this postmorterm stage MRI showed that the whole brainstems was swelling, with mixed MR signal uneven prolonged T1 and T2 signal. This information has helped us to understand the pathology for development of brainstem encephalitis, which results in the deterioration of the situation and eventual death. It was really a pity that these two cases did not undergo autopsy. Regrettably, if we had compared MRI findings from these two cases with autopsy from the existing study, we would have found consistency in both examinations[17].

The long-term MRI follow-up study demonstrated that 5 of the 31 cases had positive MRI results. From the morphological view, sagittal MR images (see Fig 9) demonstrated that the lesion narrowed with hypo-intensive signal on T1WI, and hyper-intensive signal on both T2WI and FLAIR in 3 of the cases. This MRI finding suggested that it was gliosis repairmen rather than encephalomalacia. The other 2 cases were encephalomalacia changes. These 5 patients had neurological sequelae, such as irregular breath and involuntary extremity tremor during sleeping at night. This would indicate that the permanent tissue destroy of brainstem and cause functional defect. Other neurological sequelae, such as respirator-dependent and V, VI, VII, IX nerves dysfunctions were also reported by previous study[4].

To sum up, there are three main reasons for MRI study of brainstem encephalitis induced. Firstly, a clear image of the brainstem is very important to physicians, to severity of brainstem encephalitis. Secondary, MRI provides valuable information for prognosis of this disease. For example, if you see the patient’s MRI meet type A of convalescence stage, the patient is likely to have brainstem dysfunction. Finally, the follow-up MRI study is also necessary for those who have Neurological sequelae.

### 4.3 Limitations

There are several limitations in this study that need to be declared. Firstly, all patients enrolled in this study were from PICU, and therefore, some patients with minimal brainstem encephalitis were excluded methodologically. Secondly, due to the objective condition restriction, only 31 subjects were randomly called to do the follow up MRI exams. Thirdly, the two cases with postmortem MRI did not underwent autopsy, and so the comparison study between MRI and pathology cannot be achieved. Finally, as this study only focuses on the primary lesion with follow up MRI scans, other lesions in the brain are not examined and discussed. While the use of visual analysis has been practical and successful in evaluation of diseases related to EV71, there may exist the issue of inconsistency when tuning the range of intensity values for the MR image. As such, an intensity histogram analysis for the T1WI and T2WI and derivation of their statistical properties may be useful.

## 5. Conclusion

In summary, from this study, we have provided enhanced and useful information for the MRI features of brainstem encephalitis induced by EV71 that correspond with clinical features apart from common practice established by previous reports. Furthermore, we established a classification scheme that summarizes all of our discovered types of features based on the MRI scans at the four different stages of the disease. Novel MRI findings of this study include Type IV in acute stage presenting as normal on T1WI, low intensity on T2WI, with enhancement, and whole brainstem involved in post-mortem stage. Furthermore, post-contrast can reveal the lesion missed by plain MRI scan. Clinically, radiologists should be aware of all types of MRI characters of brainstem encephalitis induced by EV71. MRI is a useful and effective modality both for diagnosis and follow-up evaluation.

## Supporting Information

S1 File This file (PDF format) contains *STROBE_checklist_cohort* for this paper. (PDF)
